# Association between uric acid to high-density lipoprotein cholesterol ratio and moderate-to-severe perivascular spaces burden: a retrospective cross-sectional study

**DOI:** 10.3389/fneur.2025.1609395

**Published:** 2025-08-01

**Authors:** Jie Lin, Jieying Zhuang, Qian Luo, Ruiyan Xiao, Huijuan Wang, Xudong Yang, Jiangping Cai

**Affiliations:** Department of Neurology, The First Hospital of Quanzhou Affiliated to Fujian Medical University, Quanzhou, Fujian, China

**Keywords:** perivascular spaces, uric acid to high-density lipoprotein cholesterol ratio, inflammation, biomarkers, cerebral small vessel disease

## Abstract

**Background:**

Perivascular spaces (PVS) are critical for waste clearance in the central nervous system and are implicated in various neurological disorders. The uric acid to high-density lipoprotein cholesterol ratio (UHR) is a novel inflammatory-metabolic marker, but its association with PVS burden remains unexplored. This study investigated the association between UHR and moderate-to-severe PVS burden.

**Methods:**

A retrospective cross-sectional analysis was conducted on 808 patients between 2022 and 2024. UHR levels were calculated and categorized into quartiles. PVS burdens in the basal ganglia and centrum semiovale regions were assessed. Logistic regression, correlation, subgroup, and restricted cubic splines analyses were performed to evaluate the relationship between UHR and PVS burden. The net reclassification index (NRI) and integrated discrimination improvement (IDI) were calculated. Additionally, sensitivity analyses were performed to validate the robustness of the findings.

**Results:**

UHR was significantly higher in patients with moderate-to-severe PVS burden compared to those with none-to-mild PVS burden (*p* < 0.001). Multivariate logistic regression revealed a positive, dose-dependent relationship between UHR and moderate-to-severe PVS burden (OR = 1.07, 95% CI: 1.03–1.12, *p* < 0.001). This association was consistent across the basal ganglia and centrum semiovale regions. Subgroup analyses suggested a consistent association across various subgroups, with LDL-C levels affecting the UHR-PVS relationship. Incorporating UHR into predictive models significantly improved the identification of moderate-to-severe PVS burden (NRI = 0.310, IDI = 0.013, *p* < 0.001).

**Conclusion:**

Our findings indicate a significant association between UHR levels and moderate-to-severe PVS burden, demonstrating potential implications for PVS risk assessment and management.

## 1 Introduction

Perivascular spaces (PVS) are fluid-filled tubular channels distributed along the course of cerebral blood vessels and serve as a critical pathway for the clearance of metabolic waste in the central nervous system (CNS) (1, 2). The recently proposed “glymphatic-like system” theory suggests that PVS plays a vital role in maintaining the homeostasis of the CNS microenvironment by facilitating the transport and exchange of cerebrospinal fluid and interstitial fluid (3). PVS has been associated with various neurological disorders, particularly cerebrovascular diseases. For example, PVS may reflect the early stage of cerebral small vessel disease. Many studies have demonstrated a substantial association between PVS and an increased risk of hemorrhagic and ischemic stroke, dementia, cerebral amyloid angiopathy (CAA), etc. (4–6). Furthermore, abnormal PVS distributions have been observed in patients with neurodegenerative diseases such as Alzheimer’s disease and Parkinson’s disease (7, 8). Additionally, there is a potential association between PVS and neuroinflammatory demyelinating diseases, including multiple sclerosis and neuromyelitis optica spectrum disorders (9, 10). Risk factors for PVS include traditional vascular risk factors such as age, hypertension, diabetes, smoking, and dyslipidemia. Moreover, chronic neuroinflammation and genetic susceptibility also contribute to the development of PVS (5, 11–14). However, the mechanisms underlying PVS and its causal relationships with neurological disorders remain poorly understood and necessitate further investigation.

Uric acid (UA) is the final product of human purine metabolism, primarily produced in the liver. It may exhibit complex dual biological effects, functioning as both an antioxidant and a pro-oxidant (15). Notably, recent studies have also demonstrated that UA can induce neuroinflammation (16) and promote brain atrophy (17), suggesting its involvement in PVS. High-density lipoprotein cholesterol (HDL-C) exerts a protective role in demyelinating nerve diseases by reducing blood–brain barrier (BBB) permeability or mitigating microglial activation (18, 19). However, excessively high HDL-C levels (>2.2 mmol/L) can promote oxidative stress and inflammatory responses (20), which are the mechanisms underlying PVS. The uric acid to high-density lipoprotein cholesterol ratio (UHR) overcomes the limitations of a single indicator and facilitates a more comprehensive and multi-faceted assessment by combining the effects of UA and HDL-C (21). It can more accurately predict the risk of diseases such as coronary artery stenosis and abdominal aortic aneurysm (22, 23). Studies have demonstrated that UHR is associated with cardiovascular diseases such as arteriosclerosis (24), coronary artery disease (25), hypertension (26), myocardial infarction (27), and atrial fibrillation (28). An increase in UHR is related to an increased risk of adverse events such as stroke in patients with diabetes (29) and coronary heart disease (30). Moreover, the all-cause mortality and cardiovascular mortality of individuals may increase with the increase of UHR, indicating a non-linear association (31, 32). Additionally, UHR is also correlated with vascular risk factors such as diabetes (33), insulin resistance (34), non-alcoholic fatty liver disease (35), chronic kidney disease (36), and metabolic syndrome (37). The extensive relationship between UHR and these diseases highlights its value in the risk assessment of cardiovascular and cerebrovascular diseases. However, the association between UHR and PVS remains inadequately explored.

This study aims to investigate the association between UHR and the burden of PVS for the first time and further assess the potential dose–response relationship. Our findings may provide new evidence for the role of metabolic-inflammatory regulation in the formation of PVS.

## 2 Methods

### 2.1 Ethics statement

This study was approved by the Ethics Committee of the Quanzhou First Hospital Affiliated with Fujian Medical University. All participants signed written informed consent.

### 2.2 Study participants

This study is a retrospective analysis involving 808 patients with cerebral small vessel disease who visited the Department of Neurology at Quanzhou First Hospital Affiliated with Fujian Medical University from February 2022 to September 2024. All patients underwent a 3.0 T brain MRI scan, routine blood tests, and biochemical evaluations. Inclusion criteria: (1) patients aged from 36 to 85 years; (2) Brain MRI sequences included at least T1-weighted imaging (T1WI), T2-weighted imaging (T2WI), Fluid Attenuated Inversion Recovery (FLAIR), and Diffusion Weighted Imaging (DWI). (3) Patients had their biochemical indexes tested through fasting blood samples within 24 h of hospitalization and underwent the brain MRI examination within 72 h. Exclusion criteria: (1) Patients with a history of large vessel occlusion or significant cerebral infarction that complicated the diagnosis of cerebral small vessel disease; (2) Patients with severe stenosis or occlusion of large intracranial vessels as shown on CT angiography or digital subtraction angiography; (3) Patients with MRI images of poor quality, contraindications for MRI scanning, or difficulties in evaluating enlarged PVS, such as having an implanted pacemaker, metal plates, dentures, or other metallic devices; suffering from claustrophobia; or being unable to cooperate; (4) Patients with severe systemic inflammatory conditions (such as hematological disorders, history of surgeries or severe trauma, active infections, or poisoning) within the past 2 weeks; (5) Patients who had taken medications that may affect UA levels, such as immunosuppressants, glucocorticoids, thyroid hormones, sex hormones, xanthine oxidase inhibitors (e.g., febuxostat, allopurinol), or uricosuric drugs (e.g., benzbromarone) within the past 2 weeks; (6) Patients with acute subarachnoid hemorrhage, a history of cerebrovascular malformation or subarachnoid hemorrhage due to an aneurysm, or an untreated aneurysm (diameter > 3 mm); (7) Patients with concurrent malignant tumors or severe liver and kidney diseases; (8) Patients with neurodegenerative diseases or significant non-vascular white matter lesions; (9) Patients with nervous system infections or metabolic-related diseases; (10) Patients with a history of traumatic brain injury or craniocerebral surgery; (11) Patients with incomplete clinical data.

### 2.3 Data collection

The baseline clinical data were collected, including gender, age, height, weight, smoking history, drinking history, and history of hypertension, diabetes, coronary heart disease, stroke, and dyslipidemia. Moreover, information on the use of antihypertensive, hypoglycemic, antiplatelet, lipid-lowering, and anticoagulant medications was recorded. Additionally, the data on triglyceride (TG), total cholesterol (TC), low-density lipoprotein cholesterol (LDL-C), HDL-C, Albumin (ALB), homocysteine (HCY), UA, creatinine (Cr), and high-sensitivity C-reactive protein (hs-CRP) were also collected. UHR was calculated as the ratio of UA to HDL-C and was classified into four quartiles: first quartile (Q1): UHR ≤ 9.48; second quartile (Q2): 9.48 < UHR ≤ 12.88; third quartile (Q3): 12.88 < UHR ≤ 17.22; and fourth quartile (Q4): UHR > 17.22.

### 2.4 MRI scanning and image analysis

All enrolled patients underwent a 3.0 T plain brain MRI scan (Signa, GE Healthcare, Milwaukee, WI, United States). The imaging sequences included T1WI, T2WI, FLAIR, and DWI. The brain MRI images of the patients were evaluated according to the Standards for Reporting Vascular Changes on Neuroimaging (STRIVE) (38). The burden of PVS was assessed by two trained neurologists who were blinded to the clinical information of all study participants. In cases of disagreement between the two neurologists, a senior neurologist made the final judgment. Furthermore, non-pathological PVS was excluded from the assessment.

Enlarged PVS were scored based on T1WI, T2WI, and FLAIR sequences. The lesions presented as round, oval, or linear imaging manifestations with diameters of 1–3 mm, exhibiting hypointensity on T1WI, hyperintensity on T2WI (similar signal intensity to cerebrospinal fluid), hypointensity on FLAIR, and no diffusion restriction on DWI. Typically, these lesions displayed clear boundaries, no contrast enhancement, and no space-occupying effects.

This study focused on enlarged PVS in the basal ganglia region (BG-PVS) and the centrum semiovale region (CSO-PVS). The number of PVS in one hemisphere of the most severely affected layer in different brain regions was counted and graded according to the following criteria (39, 40): Grade 0 = no PVS, Grade 1 ≤ 10 PVS, Grade 2 = 11–20 PVS, Grade 3 = 21–40 PVS, and Grade 4 > 40 PVS. Based on this grading, CSO-PVS and BG-PVS were classified into none-to-mild (PVS ≤ 10/region) and moderate-to-severe (PVS > 10/region) groups. This classification of using a cutoff value of 10 for PVS was recommended due to the skewness in the grading of BG-PVS and CSO-PVS (41, 42). Moreover, this was further supported by the indication that ≤10 PVS has no clinical significance (43). Additionally, patients with more than 10 enlarged PVS are at significantly increased risk for conditions such as dementia and small vessel disease (4–6, 39, 44). The severity of PVS was classified into none-to-mild PVS or moderate-to-severe PVS based on the presence of moderate-to-severe BG-PVS and/or moderate-to-severe CSO-PVS.

### 2.5 Statistical analysis

All statistical analyses were conducted using R software (R version 4.3.1). The Shapiro–Wilk normality test was performed to assess the distribution of quantitative data. Non-normally distributed data are presented as the median and interquartile range (IQR). Categorical variables are described by number and percentage [*n* (%)]. For group comparisons, the Kruskal-Wallis test, Mann–Whitney U test, chi-squared test, or Fisher’s exact test was selected based on the data type and distribution characteristics. Spearman correlation analysis was conducted to explore potential correlations among various clinical indicators, with the results presented in a heatmap.

Univariate logistic regression analysis was performed to screen for significant factors influencing the PVS burden, with a screening threshold set at *p* < 0.05. Subsequently, the collinearity of the variables was assessed using the variance inflation factor (VIF). Variables with a VIF value less than 5 were included in the multivariate logistic regression model for further verification. Notably, TC had strong collinearity with LDL-C (VIF = 8.041). LDL-C was retained in the multivariable analysis due to its established role in cardiovascular risk assessment and its specific relevance to cerebrovascular health. In contrast, TC was excluded because it does not provide additional unique information beyond that offered by LDL-C. Additionally, because the UHR includes both UA and HDL-C, these variables were not considered confounding factors in the multivariate analysis. The influencing factors for PVS burden were determined via the multivariate logistic regression analysis.

Given the non-normal distribution characteristics of the UHR, the patients were categorized based on UHR quartile levels, and a multivariate model of categorical variables was constructed to analyze the association between UHR at different levels and the burden of moderate-to-severe PVS, as well as to explore potential dose–response relationships. Specifically, Model 1 included no adjustments. In Model 2, demographic factors and personal history (age, gender, smoking history, and drinking history) were accounted for. Model 3 was constructed based on Model 2, and the additional factors of previous medical history (hypertension, diabetes, coronary heart disease, stroke, and dyslipidemia) and laboratory indicators (LDL-C, ALB, HCY, Cr, and high-hs-CRP) were adjusted. Model 4 was based on Model 3, and the medication use (antihypertensive, hypoglycemic, antiplatelet, and lipid-lowering drugs) was further adjusted. A trend test was conducted by substituting the median UHR for each group as a continuous variable in the model to calculate the *p* value. Restricted Cubic Splines (RCS) were selected to evaluate the potential non-linear relationship between UHR and the burden of moderate-to-severe PVS due to their flexibility and effectiveness in modeling such relationships while maintaining interpretability. Based on the Akaike Information Criterion, the number of knots was set to four at the 5th, 35th, 65th, and 95th percentiles, with the 50th percentile serving as the reference point.

The specificity of the spatial distribution between UHR and the burden of moderate-to-severe PVS was further assessed. Subgroup analysis was conducted based on age, gender, smoking history, drinking history, history of hypertension, HCY, ALB, LDL-C, and hs-CRP levels. The subgroup differences in the association between UHR and the burden of moderate-to-severe PVS were evaluated. Interaction analysis assessed the influence of subgroup factors on this association, with results depicted in a forest plot. After incorporating UHR into the basic model, the Net Reclassification Index (NRI) and Integrated Discrimination Improvement Index were calculated to assess the predictive incremental effect of UHR. To account for the right-skewed distribution of UHR and the potential influence of previous lipid-lowering drug treatment on UHR levels, sensitivity analyses were conducted. This included: (1) a logarithmic transformation of UHR with base 2 (log2UHR transformation); and (2) the exclusion of participants who had previously received lipid-lowering drug treatment (*n* = 123). A two-tailed *p* value < 0.05 was considered statistically significant.

## 3 Results

### 3.1 Baseline characteristics

A total of 808 patients were included in this study, with a median age of 62 years (IQR: 51–70). Among these patients, 457 (56.56%) were male. The cohort comprised 451 patients (55.82%) with hypertension, 180 (22.28%) with diabetes, 53 (6.56%) with coronary heart disease, 97 (12.00%) with a history of stroke, and 408 (50.50%) with dyslipidemia. Based on the severity of PVS, patients were classified into the none-to-mild PVS group (505 cases) and the moderate-to-severe PVS group (303 cases). Their baseline characteristics are presented in Table 1. The none-to-mild PVS group had a median age of 56 years (IQR: 48–66) and included 255 male patients (50.50%), with a median UHR level of 11.65 (IQR: 8.77–15.80). In contrast, the moderate-to-severe PVS group had 303 patients, with a median age of 67 years (IQR: 62–74), 202 (66.67%) of whom were male, and a median UHR of 14.53 (IQR: 10.94–19.41). The UHR level in the moderate-to-severe PVS group was significantly higher than that in the none-to-mild PVS group (*p* < 0.001). Furthermore, significant differences were observed between the two groups regarding age, gender, smoking history, drinking history, and the presence of comorbidities, including hypertension, diabetes, coronary heart disease, stroke, and dyslipidemia. Differences in the use of antihypertensive, hypoglycemic, antiplatelet, and lipid-lowering medications, as well as laboratory parameters such as TG, TC, LDL-C, HDL-C, ALB, HCY, Cr, and UA, were also statistically significant (*p* < 0.05). However, no significant differences were found regarding body mass index (BMI) and the use of anticoagulant drugs (*p* > 0.05).

**Table 1:** Baseline characteristics of the none-to-mild PVS group and the moderate-to-severe PVS group.

Variables	None-to-mild PVS group (*n* = 505)	Moderate-to-severe PVS group (*n* = 303)	*p*
Age, years	56 (IQR: 48–66)	67 (IQR: 62–74)	<0.001
Male, *n* (%)	255 (50.50)	202 (66.67)	<0.001
BMI (kg/m^2^)	23.42 (IQR: 21.97–24.75)	23.53 (IQR: 22.31–24.74)	0.220
Smoking history, *n* (%)	102 (20.20)	128 (42.24)	<0.001
Drinking history, *n* (%)	43 (8.51)	59 (19.47)	<0.001
Comorbidities			
Hypertension, *n* (%)	211 (41.78)	240 (79.21)	<0.001
Diabetes, *n* (%)	72 (14.26)	108 (35.64)	<0.001
Coronary heart disease, *n* (%)	15 (2.97)	38 (12.54)	<0.001
Stroke, *n* (%)	28 (5.54)	69 (22.77)	<0.001
Dyslipidemia, *n* (%)	220 (43.56)	188 (62.05)	<0.001
Medication use			
Antihypertensive drugs, *n* (%)	109 (21.58)	134 (44.22)	<0.001
Hypoglycemic drugs, *n* (%)	35 (6.93)	52 (17.16)	<0.001
Antiplatelet drugs, *n* (%)	28 (5.54)	55 (18.15)	<0.001
Anticoagulant drugs, *n* (%)	2 (0.40)	3 (0.99)	0.562
Lipid-lowering drugs, *n* (%)	54 (10.69)	69 (22.77)	<0.001
Blood biochemical indicators			
TG, mmol/L	1.19 (IQR: 0.85–1.70)	1.28 (IQR: 0.94–1.86)	0.043
TC, mmol/L	4.97 (IQR: 4.26–5.76)	4.75 (IQR: 3.79–5.55)	0.002
LDL-C, mmol/L	3.22 (IQR: 2.65–3.81)	3.03 (IQR: 2.26–3.70)	<0.001
HDL-C, mmol/L	1.19 (IQR: 1.03–1.43)	1.07 (IQR: 0.92–1.27)	<0.001
ALB, g/L	39.60 (IQR: 37.50–42.10)	38.80 (IQR: 36.50–41.05)	<0.001
HCY, μmol/L	9.20 (IQR: 7.00–11.80)	12.60 (IQR: 9.85–16.15)	<0.001
Cr, μmol/L	65.50 (IQR: 54.20–78.00)	71.30 (IQR: 61.65–85.85)	<0.001
hs-CRP, mg/L	0.56 (IQR: 0.50–2.77)	2.40 (IQR: 0.55–5.00)	<0.001
UA, μmol/L	325.00 (IQR: 269.00–390.00)	369.00 (IQR: 298.50–444.50)	<0.001
UHR (%)	11.65 (IQR: 8.77–15.80)	14.53 (IQR: 10.94–19.41)	<0.001

Non-normally distributed data are presented as the median and IQR. Categorical variables are described by number and percentage [*n* (%)]. IQR, interquartile range; PVS, perivascular spaces; BMI, body mass index; TG, triglyceride; TC, total cholesterol; LDL-C, low-density lipoprotein cholesterol; HDL-C, high-density lipoprotein cholesterol; ALB, albumin; HCY, homocysteine; UA, urine acid; Cr, creatinine; hs-CRP, high-sensitivity C-reactive protein; UHR, uric acid to high-density lipoprotein cholesterol ratio.

### 3.2 Correlation analysis among various clinical indicators

**Figure 1:**
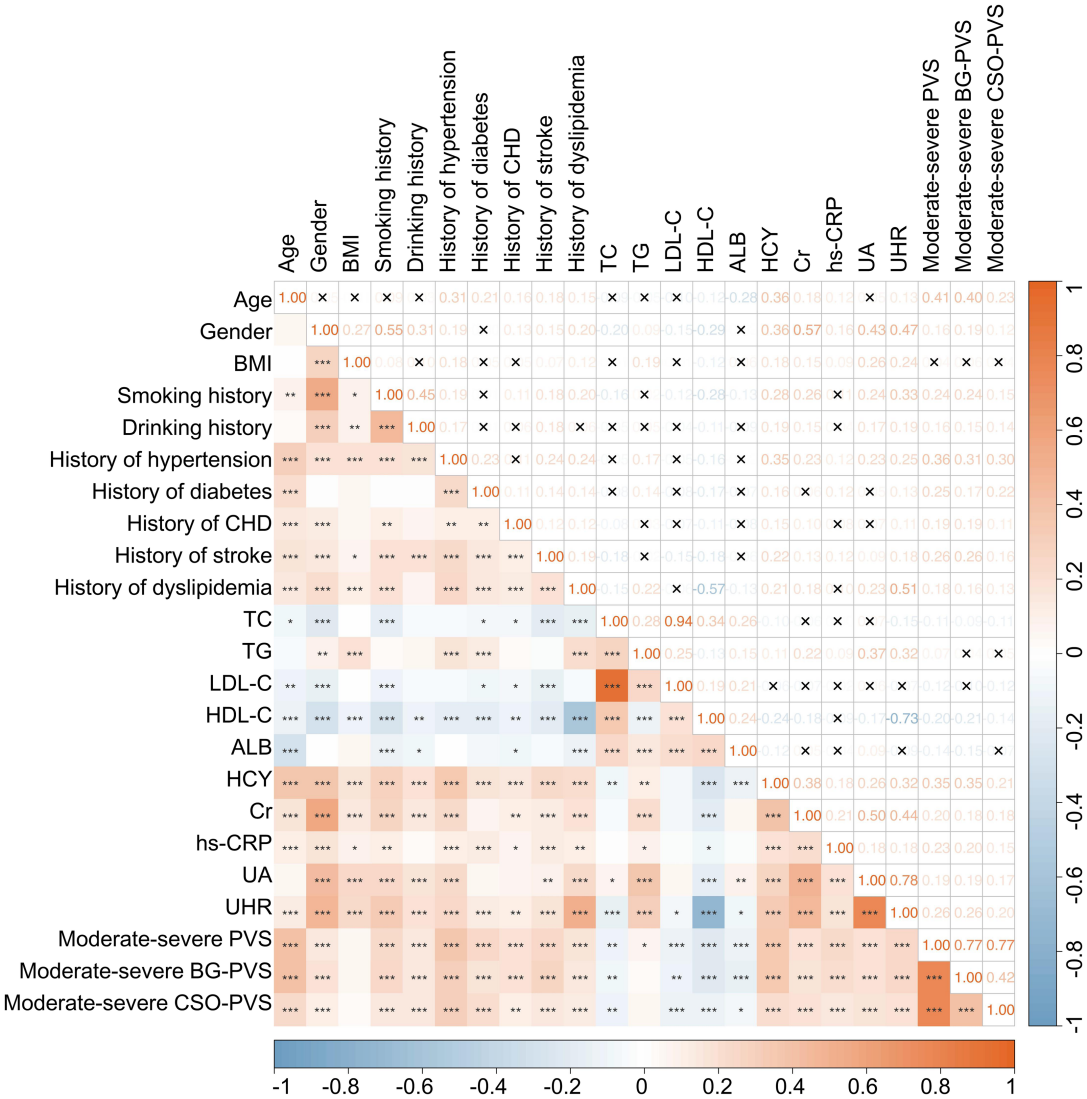
Heatmap illustrating the correlation matrix among various clinical indicators. ^*^p < 0.05, ^**^p < 0.01, ^***^p < 0.001, ^×^*p* > 0.05.

A Spearman correlation analysis was performed to analyze the potential correlations among various clinical indicators. The results demonstrated that moderate-to-severe PVS burden and UHR exhibited significant correlations with several indicators (Figure 1). Specifically, the moderate-to-severe PVS burden showed a moderate positive correlation with age (rs = 0.41, *p* < 0.001) and significant weak correlations with gender (rs = 0.16, *p* < 0.001), smoking history (rs = 0.24, *p* < 0.001), history of hypertension (rs = 0.36, *p* < 0.001), history of diabetes (rs = 0.25, *p* < 0.001), history of stroke (rs = 0.26, *p* < 0.001), HDL-C (rs = −0.20, *p* < 0.001), HCY (rs = 0.35, *p* < 0.001), Cr (rs = 0.20, *p* < 0.001), hs-CRP (rs = 0.23, *p* < 0.001), and UHR (rs = 0.26, *p* < 0.001). In contrast, no significant correlation was observed with BMI (*p* > 0.05). Furthermore, in addition to strong correlations with UA (rs = 0.78, *p* < 0.001) and HDL-C (rs = −0.73, *p* < 0.001), UHR exhibited a moderate correlation with gender (rs = 0.47, *p* < 0.001), dyslipidemia (rs = 0.51, *p* < 0.001), and Cr (rs = 0.44, *p* < 0.001). Additionally, UHR had weak correlations with BMI (rs = 0.24, *p* < 0.001), smoking history (rs = 0.33, *p* < 0.001), hypertension (rs = 0.25, *p* < 0.001), TG (rs = 0.32, *p* < 0.001), and HCY (rs = 0.32, *p* < 0.001).

### 3.3 Logistic regression analysis of influencing factors of the PVS burden

The multivariate logistic regression analysis revealed that, after adjusting for age, gender, smoking history, drinking history, hypertension, diabetes, coronary heart disease, stroke, dyslipidemia, LDL-C, ALB, HCY, Cr, hs-CRP, and the use of antihypertensive, hypoglycemic, antiplatelet, and lipid-lowering medications, the UHR was independently associated with a moderate-to-severe PVS burden (odds ratio (OR) = 1.07, 95% confidence interval (CI): 1.03–1.12, *p* < 0.001) (Table 2). Additionally, the analysis identified age (OR = 1.07, 95% CI: 1.05–1.09, *p* < 0.001), smoking history (OR = 1.98, 95% CI: 1.22–3.21, *p* = 0.005), a history of hypertension (OR = 2.95, 95% CI: 1.86–4.66, *p* < 0.001), a history of diabetes (OR = 2.15, 95% CI: 1.26–3.67, *p* = 0.005), a history of coronary heart disease (OR = 2.26, 95% CI: 1.08–4.71, *p* = 0.030), a history of stroke (OR = 1.84, 95% CI: 1.04–3.25, *p* = 0.037), and HCY (OR = 1.02, 95% CI: 1.01–1.05, *p* = 0.045) as additional independent risk factors for the PVS burden (Table 2).

**Table 2:** Univariate and multivariate logistic regression analysis of factors influencing PVS burden.

Variables	Univariate logistic regression		Multivariate logistic regression^a^	
	OR (95% CI)	*p*	OR (95% CI)	*p*
Age	1.09 (1.07–1.11)	<0.001	1.07 (1.05–1.09)	<0.001
Gender	1.96 (1.46–2.64)	<0.001		
BMI	2.89 (2.11–3.96)	<0.001	1.98 (1.22–3.21)	0.005
Smoking history	2.60 (1.70–3.96)	<0.001		
Drinking history	5.31 (3.82–7.38)	<0.001	2.95 (1.86–4.66)	<0.001
History of hypertension	3.33 (2.36–4.69)	<0.001	2.15 (1.26–3.67)	0.005
History of diabetes	4.68 (2.53–8.67)	<0.001	2.26 (1.08–4.71)	0.030
History of coronary heart disease	5.02 (3.15–8.01)	<0.001	1.84 (1.04–3.25)	0.037
History of stroke	2.12 (1.58–2.83)	<0.001		
Antihypertensive drugs	2.88 (2.11–3.93)	<0.001		
Hypoglycemic drugs	2.78 (1.76–4.39)	<0.001		
Antiplatelet drugs	3.78 (2.34–6.11)	<0.001		
Lipid-lowering drugs	2.46 (1.67–3.64)	<0.001		
TC^b^	0.84 (0.75–0.95)	0.005		
TG	1.08 (0.93–1.26)	0.313		
LDL-C	0.76 (0.65–0.89)	<0.001		
ALB	0.94 (0.91–0.98)	0.002		
HCY	1.09 (1.06–1.13)	<0.001	1.02 (1.01–1.05)	0.045
Cr	1.01 (1.01–1.01)	0.004		
hs-CRP	1.03 (1.01–1.04)	0.001		
UHR	1.10 (1.07–1.13)	<0.001	1.07 (1.03–1.12)	<0.001

^a^Adjusted for age, gender, smoking history, drinking history, history of hypertension, history of diabetes, history of coronary heart disease, history of stroke, history of dyslipidemia, LDL-C, ALB, HCY, Cr, hs-CRP, antihypertensive drugs, hypoglycemic drugs, antiplatelet drugs, and lipid-lowering drugs. ^b^TC was not included in the multivariate regression model due to its high collinearity with LDL-C (VIF = 8.041). PVS, perivascular spaces; BMI, body mass index; TG, triglyceride; TC, total cholesterol; LDL-C, low-density lipoprotein cholesterol; HDL-C, high-density lipoprotein cholesterol; ALB, albumin; HCY, homocysteine; UA, urine acid; Cr, creatinine; hs-CRP, high-sensitivity C-reactive protein; UHR, uric acid to high-density lipoprotein cholesterol ratio; OR, odds ratio; 95% CI, 95% confidence interval.

### 3.4 Multivariate logistic regression analysis of the relationship between UHR and the moderate-to-severe PVS burden

A multivariate logistic regression analysis was conducted to further explore the relationship between UHR and the moderate-to-severe PVS burden (Table 3). The results indicated that an increase in UHR significantly elevated the risk of moderate-to-severe PVS, both as a continuous variable and as categorized into quartiles (*p* < 0.001). In Model 1, before adjusting for any variables, the OR for UHR was 1.10 (95% CI: 1.07–1.13, *p* < 0.001). After gradually adjusting for demographic characteristics and personal history in Model 2, past medical history and biochemical indices in Model 3, and drug use in Model 4, the effect size decreased slightly but remained statistically significant (*p* < 0.001). In the fully adjusted model, compared to that in the Q1 group, the UHR levels in the Q2 group (OR = 1.86, 95% CI: 1.08–3.19, *p* = 0.025), the Q3 group (OR = 1.90, 95% CI: 1.07–3.37, *p* = 0.028), and the Q4 group (OR = 2.64, 95% CI: 1.41–4.94, *p* = 0.002) were associated with an increasing risk of moderate-to-severe PVS (P-trend = 0.008).

**Table 3:** Multivariate logistic regression analysis of the association between UHR and the burden of moderate-to-severe PVS burden.

Models	Model 1	Model 2	Model 3	Model 4
	OR (95% CI)	OR (95% CI)	OR (95% CI)	OR (95% CI)
Continuous variable model				
UHR	1.10 (1.07–1.13)	1.09 (1.05–1.12)	1.07 (1.03–1.12)	1.07 (1.03–1.12)
*p*	<0.001	<0.001	<0.001	<0.001
Categorical variable model				
Q1	1.00 (Reference)	1.00 (Reference)	1.00 (Reference)	1.00 (Reference)
Q2	2.15 (1.37–3.37)	2.00 (1.21–3.31)	1.86 (1.08–3.19)	1.86 (1.08–3.19)
Q3	2.98 (1.91–4.64)	2.41 (1.45–4.00)	1.94 (1.10–3.42)	1.90 (1.07–3.37)
Q4	4.61 (2.96–7.18)	3.36 (1.98–5.69)	2.74 (1.47–5.08)	2.64 (1.41–4.94)
*P* for trend	<0.001	<0.001	0.004	0.008

In Model 1, no factors were adjusted; in Model 2, age, gender, smoking history, and drinking history were adjusted; based on Model 2, in Model 3, the history of hypertension, history of diabetes, history of coronary heart disease, history of stroke, history of dyslipidemia, LDL-C, ALB, HCY, Cr, and hs-CRP were adjusted; based on Model 3, in Model 4, the use of antihypertensive drugs, hypoglycemic drugs, antiplatelet drugs, and lipid-lowering drugs was adjusted. PVS, perivascular spaces; UHR, uric acid to high-density lipoprotein cholesterol ratio; OR, odds ratio; 95% CI, 95% confidence interval.

### 3.5 The dose–response relationship between UHR and the moderate-to-severe PVS burden

**Figure 2:**
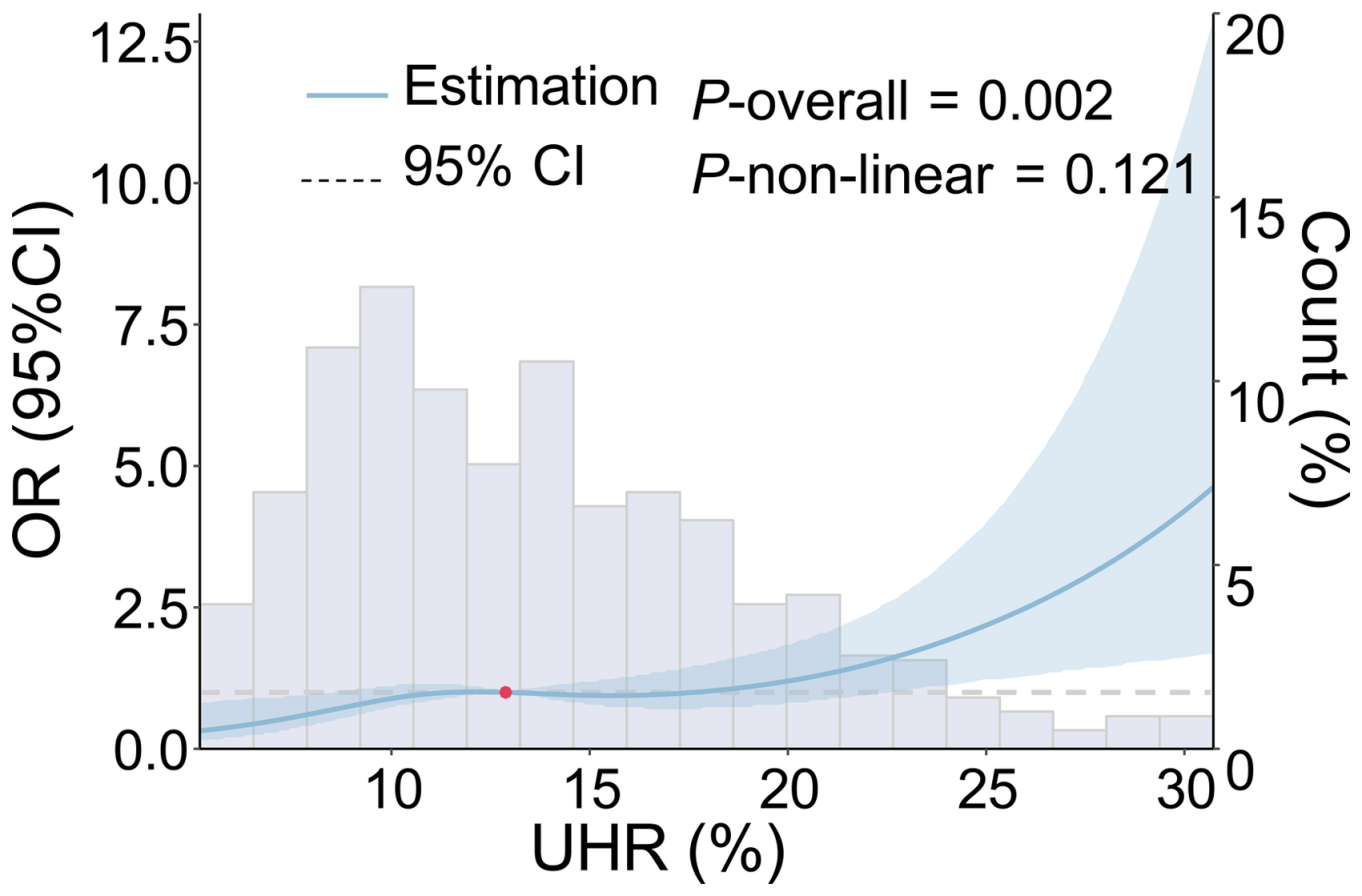
Dose–response relationship between UHR and the risk of moderate-to-severe PVS. The following factors were adjusted, including age, gender, smoking history, drinking history, history of hypertension, history of diabetes, history of coronary heart disease, history of stroke, history of dyslipidemia, LDL-C, ALB, HCY, Cr, hs-CRP, and the use of antihypertensive, hypoglycemic, antiplatelet, and lipid-lowering drugs. The blue solid line represents the OR value, the blue shadow represents the 95% CI, and the red dot represents the 50th percentile reference point.

The RCS analysis indicated that, after adjusting for age, gender, smoking history, drinking history, hypertension, diabetes, coronary heart disease, stroke, dyslipidemia, LDL-C, ALB, HCY, Cr, hs-CRP, and the use of antihypertensive, hypoglycemic, antiplatelet, and lipid-lowering drugs, a linear positive relationship was observed between UHR and the moderate-to-severe PVS burden (p = 0.002, P for non-linear test = 0.121) (Figure 2). Furthermore, as the UHR level increased, the risk of moderate-to-severe PVS increased in a dose-dependent manner.

### 3.6 Specificity of the spatial distribution between UHR and the moderate-to-severe PVS burden

#### 3.6.1 Association between UHR and the moderate-to-severe BG-PVS burden

**Figure 3:**
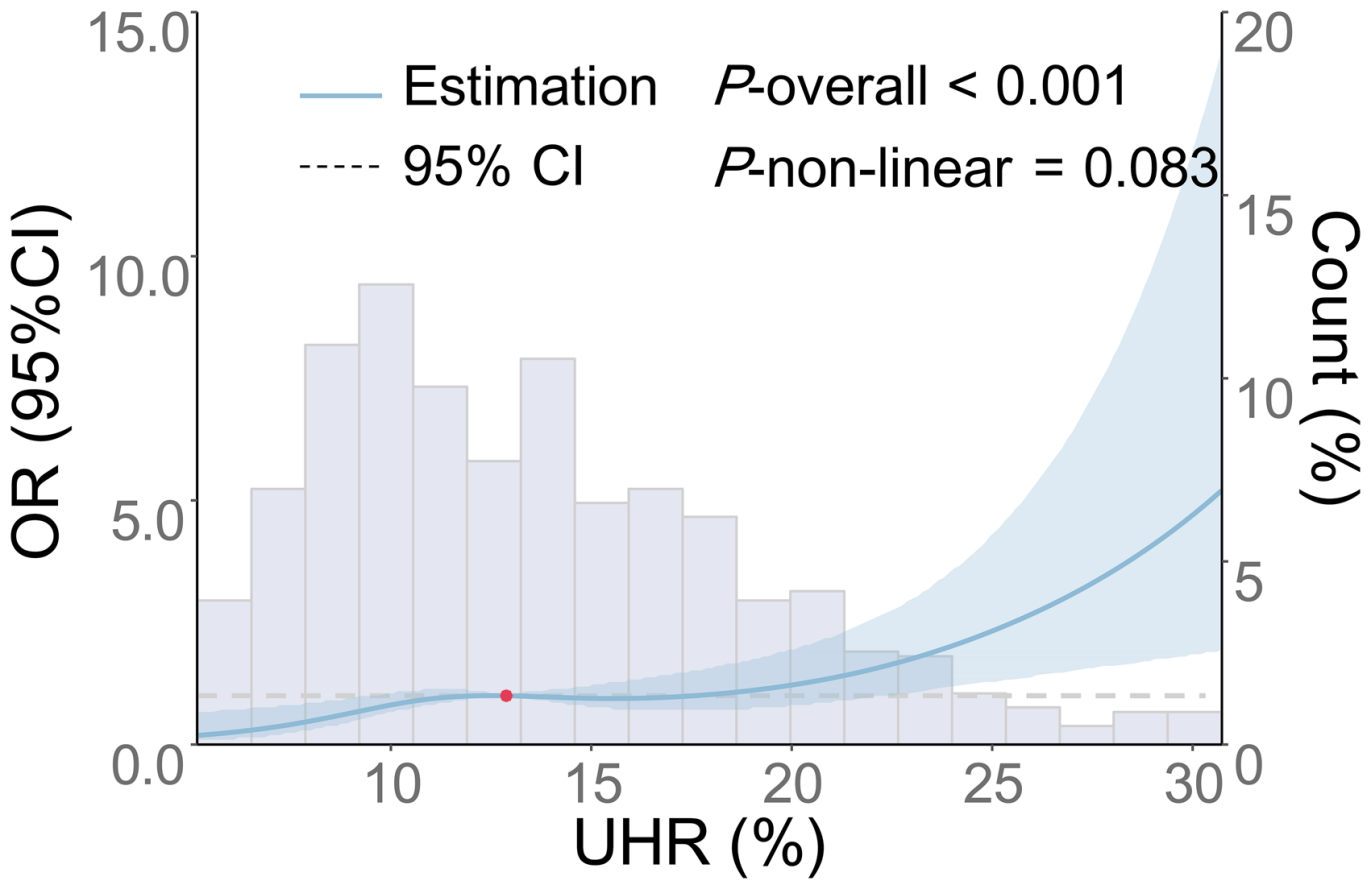
Dose–response relationship between UHR and the risk of moderate-to-severe BG-PVS. The following factors were adjusted: age, gender, smoking history, drinking history, history of hypertension, history of diabetes, history of coronary heart disease, history of stroke, history of dyslipidemia, LDL-C, ALB, HCY, Cr, hs-CRP, and the use of antihypertensive, hypoglycemic, antiplatelet, and lipid-lowering medications. The blue solid line represents the OR, the blue shaded area denotes the 95% CI, and the red dot indicates the 50th percentile reference point.

Multivariate logistic regression analysis revealed that increases in UHR were independently associated with the moderate-to-severe BG-PVS burden (*p* < 0.001), and this association remained consistent across all models (Table 4). In the fully adjusted model (Model 4), UHR was independently associated with the moderate-to-severe BG-PVS burden (OR = 1.09, 95% CI: 1.04–1.13, p < 0.001). Analysis of the categorical variable model indicated that, compared to that in the Q1 group, the UHR levels in the Q2 group (OR = 1.97, 95% CI: 1.04–3.74, *p* = 0.038), the Q3 group (OR = 2.32, 95% CI: 1.20–4.47, *p* = 0.012), and the Q4 group (OR = 3.09, 95% CI: 1.53–6.23, *p* = 0.002) were all associated with an increased risk of moderate-to-severe BG-PVS burden (*P*-trend = 0.005). Additionally, the RCS analysis was conducted to further evaluate the dose–response relationship between UHR and moderate-to-severe BG-PVS burden (Figure 3). The results demonstrated that after adjusting for confounding factors, there was a linear positive association between UHR and the moderate-to-severe BG-PVS burden (*p* < 0.001, *P* for non-linear test = 0.083).

**Table 4:** Multivariate logistic regression analysis for the association between UHR and the burden of moderate-to-severe BG-PVS.

Models	Model 1	Model 2	Model 3	Model 4
	OR (95% CI)	OR (95% CI)	OR (95% CI)	OR (95% CI)
Continuous variable model				
UHR	1.11 (1.08–1.14)	1.08 (1.05–1.12)	1.09 (1.04–1.13)	1.09 (1.04–1.13)
*p*	<0.001	<0.001	<0.001	<0.001
Categorical variable model				
Q1	1.00 (Reference)	1.00 (Reference)	1.00 (Reference)	1.00 (Reference)
Q2	2.48 (1.43–4.30)	2.19 (1.19–4.01)	1.98 (1.05–3.76)	1.97 (1.04–3.74)
Q3	3.68 (2.16–6.28)	2.74 (1.50–5.00)	2.29 (1.19–4.41)	2.32 (1.20–4.47)
Q4	5.41 (3.20–9.15)	3.39 (1.85–6.23)	3.12 (1.56–6.23)	3.09 (1.53–6.23)
*P* for trend	<0.001	<0.001	0.004	0.005

In Model 1, no factors were adjusted; in Model 2, age, gender, smoking history, and drinking history were adjusted; based on Model 2, in Model 3, the history of hypertension, history of diabetes, history of coronary heart disease, history of stroke, history of dyslipidemia, LDL-C, ALB, HCY, Cr, and hs-CRP were adjusted; based on Model 3, in Model 4, the use of antihypertensive drugs, hypoglycemic drugs, antiplatelet drugs, and lipid-lowering drugs was adjusted. PVS, perivascular spaces; BG-PVS, PVS in the basal ganglia region; UHR, uric acid to high-density lipoprotein cholesterol ratio; OR, odds ratio; 95% CI, 95% confidence interval.

#### 3.6.2 Association between UHR and the moderate-to-severe CSO-PVS burden

**Figure 4:**
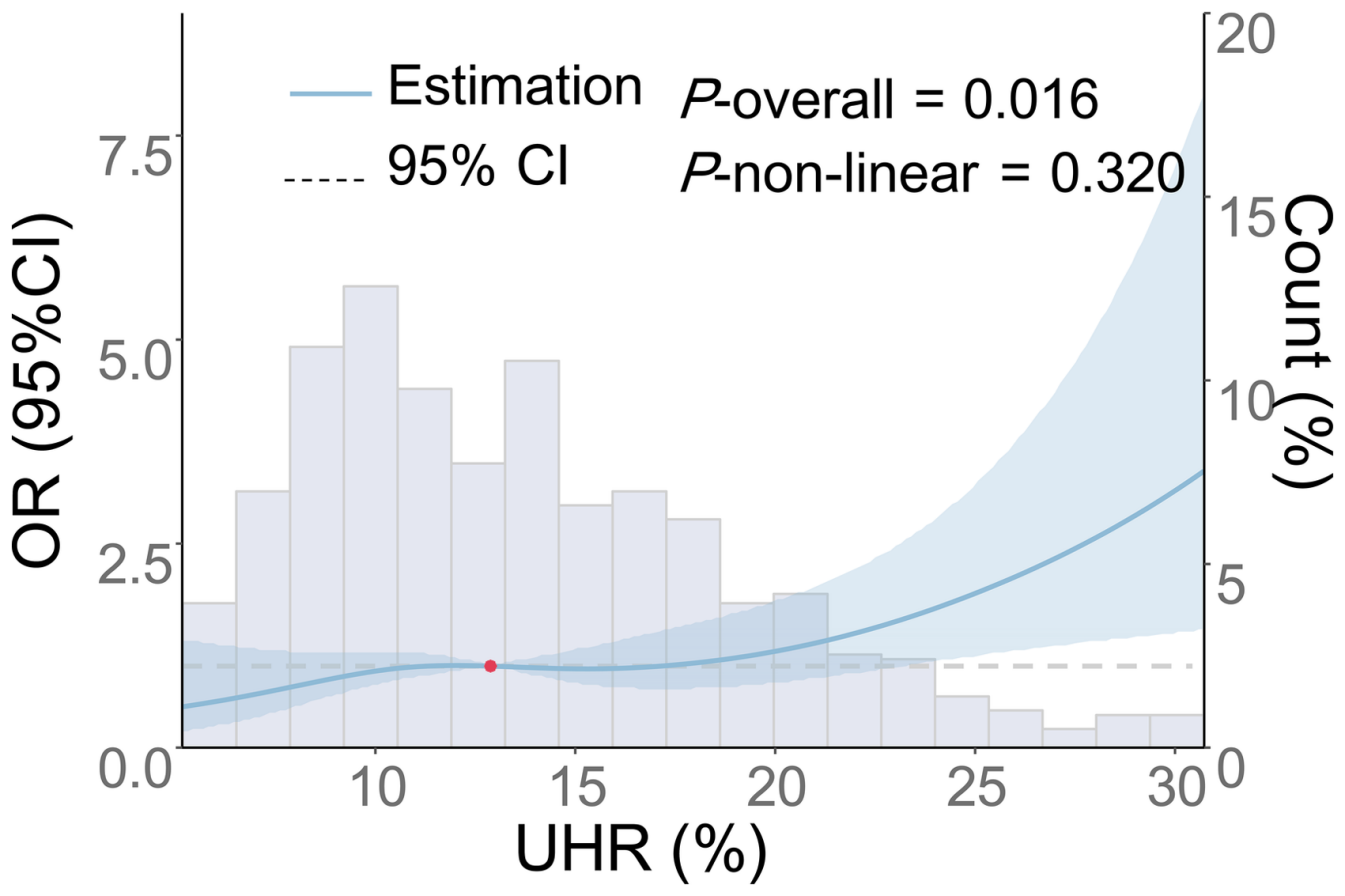
Dose–response relationship between UHR and the risk of moderate-to-severe CSO-PVS. Adjustments were made for age, gender, smoking history, drinking history, history of hypertension, diabetes, coronary heart disease, stroke, dyslipidemia, LDL-C, ALB, HCY, Cr, hs-CRP, and the use of antihypertensive, hypoglycemic, antiplatelet, and lipid-lowering medications. The blue solid line represents the OR, the blue shaded area denotes the 95% CI, and the red dot indicates the 50th percentile reference point.

As shown in Table 5, there was an independent association between the continuous variable of UHR and the risk of moderate-to-severe CSO-PVS across Models 1–4 (*p* < 0.001), as indicated by multivariate logistic regression analysis. In the fully adjusted model (Model 4), each 1-unit increase in UHR was associated with a 6% increase in the risk of moderate-to-severe CSO-PVS lesions (OR = 1.06, 95% CI: 1.02–1.10, *p* < 0.001). In the analysis of quartile categorical variables, after adjusting for all confounding factors, only the UHR level in the Q4 group, using the Q1 group as a reference, was associated with an increased burden of moderate-to-severe CSO-PVS (OR = 1.92, 95% CI: 1.03–3.58, *p* = 0.040). The UHR levels in the Q2 and Q3 groups did not show a significant association with the moderate-to-severe CSO-PVS burden (*p* > 0.05). The trend test demonstrated a significant linear increase in CSO-PVS burden with increasing quartiles of UHR (*P*-trend = 0.029). Furthermore, the dose-dependent relationship between UHR and moderate-to-severe CSO-PVS burden was analyzed using RCS analysis (Figure 4). After adjusting for confounding factors, a linear positive association was found between UHR and moderate-to-severe CSO-PVS burden (*p* = 0.016, P for non-linear test = 0.320), indicating that increases in UHR levels were dose-dependently related to the burden of moderate-to-severe CSO-PVS lesions.

**Table 5:** Multivariate logistic regression analysis for the relationship between UHR and the burden of moderate-to-severe CSO-PVS.

Models	Model 1	Model 2	Model 3	Model 4
	OR (95% CI)	OR (95% CI)	OR (95% CI)	OR (95% CI)
Continuous variable model				
UHR	1.09 (1.06–1.12)	1.07 (1.04–1.11)	1.06 (1.02–1.09)	1.06 (1.02–1.10)
*p*	<0.001	<0.001	0.003	0.003
Categorical variable model				
Q1	1.00 (Reference)	1.00 (Reference)	1.00 (Reference)	1.00 (Reference)
Q2	1.44 (0.87–2.38)	1.29 (0.76–2.18)	1.18 (0.68–2.05)	1.18 (0.68–2.06)
Q3	2.39 (1.48–3.87)	1.92 (1.15–3.23)	1.57 (0.89–2.77)	1.55 (0.88–2.75)
Q4	3.16 (1.97–5.08)	2.37 (1.39–4.03)	1.88 (1.02–3.48)	1.92 (1.03–3.58)
*P* for trend	<0.001	0.001	0.031	0.029

In Model 1, no factors were adjusted; in Model 2, age, gender, smoking history, and drinking history were adjusted; based on Model 2, in Model 3, the history of hypertension, history of diabetes, history of coronary heart disease, history of stroke, history of dyslipidemia, LDL-C, ALB, HCY, Cr, and hs-CRP were adjusted; based on Model 3, in Model 4, antihypertensive drugs, hypoglycemic drugs, antiplatelet drugs, and lipid-lowering drugs were adjusted. PVS, perivascular spaces; CSO-PVS, PVS in the centrum semiovale region; UHR, uric acid to high-density lipoprotein cholesterol ratio; OR, odds ratio; 95% CI, 95% confidence interval.

### 3.7 Subgroup analysis and interaction analysis of the association between UHR and the moderate-to-severe PVS burden

**Figure 5:**
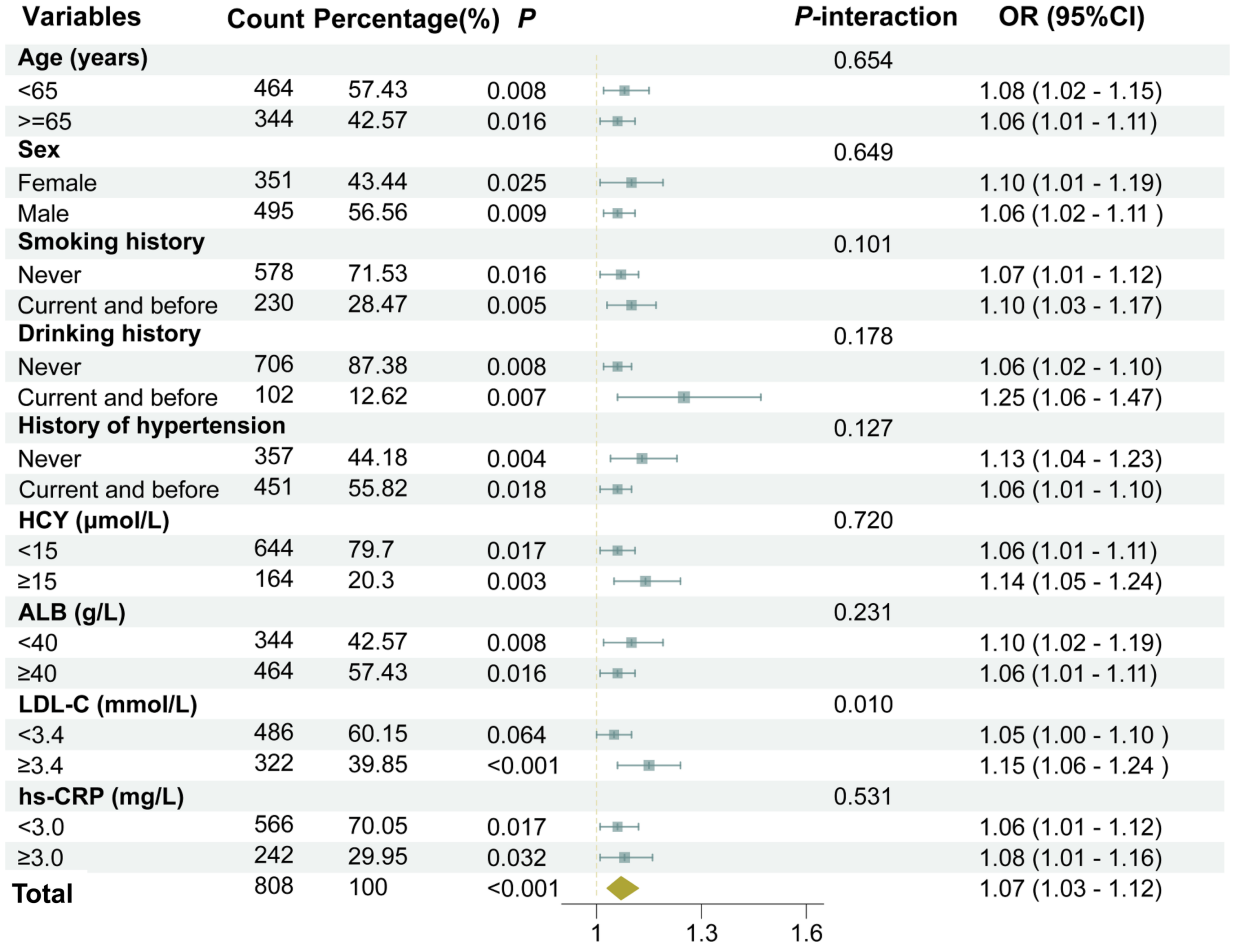
A forest plot showing the results of subgroup and interaction analyses. The analysis was adjusted for several confounding variables, including age, gender, smoking history, drinking history, history of hypertension, diabetes, coronary heart disease, stroke, dyslipidemia, as well as laboratory markers such as LDL-C, HCY, Cr, hs-CRP, and the use of medications including antihypertensive agents, hypoglycemic drugs, antiplatelet medications, and lipid-lowering drugs.

The association between UHR and the moderate-to-severe PVS burden was further assessed in various subgroups based on age (≥65 years old or <65 years old), gender (male or female), smoking history (yes or no), drinking history (yes or no), history of hypertension (yes or no), HCY (≥15 μmol or <15 μmol), ALB (≥40 g/L or <40 g/L), and hs-CRP (≥3 mg/L or <3 mg/L). The results revealed that the associations between UHR and the moderate-to-severe PVS burden were all statistically significant in these subgroups (*p* < 0.05), except for the subgroup with LDL-C levels < 3.4 mmol/L (*p* = 0.064) (Figure 5). Furthermore, the interaction analysis revealed a significant interaction effect between LDL-C and UHR (*P*-interaction = 0.010). Additionally, no obvious interaction was observed between UHR and the variables in the remaining subgroups (*P*-interaction > 0.05).

### 3.8 Efficacy of UHR in predicting moderate-to-severe PVS burden

The incremental value of UHR in predicting the moderate-to-severe PVS burden was further evaluated using the NRI and IDI. As presented in Table 6, incorporating UHR into the basic model, which included traditional risk factors and drug use, improved the performance of UHR in predicting the moderate-to-severe PVS burden. The NRI and IDI were 0.310 (95% CI: 0.169–0.451, *p* < 0.001) and 0.013 (95% CI: 0.004–0.021, *p* = 0.003), respectively. Furthermore, the NRI and IDI of UHR for the moderate-to-severe BG-PVS burden were 0.327 (95% CI: 0.172–0.482, *p* < 0.001) and 0.021 (95% CI: 0.008–0.034, *p* = 0.003), respectively; while for the moderate-to-severe CSO-PVS burden were 0.254 (95% CI: 0.099–0.410, *p* = 0.001) and 0.012 (95% CI: 0.002–0.021, *p* = 0.016), respectively.

**Table 6:** Analysis of the efficacy of UHR predicting moderate-to-severe PVS burden.

Models	NRI		IDI	
	Estimation (95% CI)	*p*	Estimation (95% CI)	*p*
Moderate-to-severe PVS				
Basic model	-	-	-	-
Basic model + UHR	0.310 (0.169–0.451)	<0.001	0.013 (0.004–0.021)	0.003
Moderate-to-severe BG-PVS				
Basic model	-	-	-	-
Basic model + UHR	0.327 (0.172–0.482)	<0.001	0.021 (0.008–0.034)	0.001
Moderate-to-severe CSO-PVS				
Basic model	-	-	-	-
Basic model + UHR	0.254 (0.099–0.410)	0.001	0.012 (0.002–0.021)	0.016

The basic model is a multivariate logistic regression model including age, gender, smoking history, drinking history, history of hypertension, history of diabetes, history of coronary heart disease, history of stroke, history of dyslipidemia, LDL-C, ALB, HCY, Cr, hs-CRP, and the use of antihypertensive, hypoglycemic, antiplatelet, and lipid-lowering drugs. PVS, perivascular spaces; BG-PVS, PVS in the basal ganglia region; CSO-PVS, PVS in the centrum semiovale region; UHR, uric acid to high-density lipoprotein cholesterol ratio; OR, odds ratio; 95% CI, 95% confidence interval.

### 3.9 Sensitivity analysis

Sensitivity analyses were conducted to assess the robustness of our findings regarding the association between UHR and PVS burden (Table 7). First, we performed a sensitivity analysis using a logarithmic transformation of UHR (log2UHR transformation). This transformation addressed the non-normal distribution characteristics of UHR data and attempted to stabilize variances. The results from this analysis demonstrated that log2UHR remained significantly associated with moderate-to-severe PVS burden (OR = 1.96, 95% CI: 1.33–2.90, *p* < 0.001). Moreover, the risks of moderate-to-severe BG-PVS and moderate-to-severe CSO-PVS burdens increased by 141% (OR = 2.41, 95% CI: 1.56–3.70, *p* < 0.001) and 69% (OR = 1.69, 95% CI: 1.16–2.48, *p* = 0.007), respectively. These findings suggest that utilizing transformed UHR values does not alter the observed risk relationship and reinforces the robustness of our conclusions regarding the impact of UHR on PVS burden.

**Table 7:** Sensitivity analysis of the relationship between UHR and moderate-to-severe PVS burden.

Sensitivity analysis	Moderate-to-severe PVS		Moderate-to-severe BG-PVS		Moderate-to-severe CSO-PVS	
	OR (95% CI)	*p*	OR (95% CI)	*p*	OR (95% CI)	*p*
Sensitivity analysis 1						
Log_2_UHR	1.96 (1.33–2.90)	<0.001	2.41 (1.56–3.70)	<0.001	1.69 (1.16–2.48)	0.007
Sensitivity analysis 2						
UHR	1.08 (1.03–1.13)	<0.001	1.10 (1.05–1.16)	<0.001	1.06 (1.01–1.10)	0.011

In sensitivity analysis 1, the logarithmic transformation of UHR with base 2 was carried out. In sensitivity analysis 2, the population that received lipid-lowering drugs (*n* = 123) was excluded. For both analyses, the adjusted factors included age, gender, smoking history, drinking history, history of hypertension, history of diabetes, history of coronary heart disease, history of stroke, history of dyslipidemia, LDL-C, ALB, HCY, Cr, hs-CRP, and the use of antihypertensive, hypoglycemic, antiplatelet, and lipid-lowering drugs. PVS, perivascular spaces; BG-PVS, PVS in the basal ganglia region; CSO-PVS, PVS in the centrum semiovale region; UHR, uric acid to high-density lipoprotein cholesterol ratio; OR, odds ratio; 95% CI, 95% confidence interval.

Additionally, we excluded patients who were on lipid-lowering medications, as these drugs are known to influence lipid levels and possibly alter the UHR. After excluding 123 patients, the associations between UHR and moderate-to-severe PVS burden (OR = 1.08, 95% CI: 1.03–1.13, *p* < 0.001), moderate-to-severe BG-PVS burden (OR = 1.10, 95% CI: 1.05–1.16, *p* < 0.001), and moderate-to-severe CSO-PVS burden (OR = 1.06, 95% CI: 1.01–1.10, *p* = 0.011) remained statistically significant, indicating that the observed relationship between UHR and PVS burden was not primarily driven by these medications.

## 4 Discussion

This study systematically investigated the association between UHR and moderate-to-severe PVS burden for the first time. The findings indicate that UHR levels in the moderate-to-severe PVS group were significantly higher than those in the none-to-mild PVS group. UHR was independently and positively correlated with moderate-to-severe PVS burden, revealing a linear dose–response relationship, which suggests that UHR may serve as a potential risk factor for moderate-to-severe PVS burden. Furthermore, UHR was associated with a linear, dose-dependent increase in the risks of moderate-to-severe BG-PVS and moderate-to-severe CSO-PVS burdens. Subgroup analyses indicated that the relationship between UHR and PVS burden remained robust across most subgroups except for the subgroup with LDL-C levels < 3.4 mmol/L. Interaction analysis suggested that LDL-C had a moderating effect on the association. Including UHR in the basic model significantly enhanced predictive performance for PVS, BG-PVS, and CSO-PVS burdens. Finally, two sensitivity analyses were conducted to further validate the robustness of the core conclusions. The results indicate a positive association between UHR and PVS burden, suggesting that UHR may serve as a potential indicator for predicting the severity of PVS.

Although there is no study on the association between UHR and PVS, the relationships between UA, HDL-C, and PVS have been widely reported. For example, a study involving 665 patients with lacunar stroke found that UA was positively correlated with both the BG-PVS burden and the CSO-PVS burden, suggesting that UA is an independent risk factor for PVS (45). Li et al. (46) indicated that UA was associated with an increased risk of PVS, identifying it as a key predictor for its occurrence. A population-based multimodal imaging study demonstrated a significant linear association between elevated UA levels and an increased risk of midbrain enlarged PVS, suggesting that UA may play a crucial role in the pathogenic mechanisms of PVS in specific brain regions (47). In contrast, the existing evidence regarding the association between HDL-C and PVS is contradictory. One study found that CSO-PVS may be positively correlated with UA and negatively correlated with HDL-C (48). A multicenter study revealed that decreased levels of apolipoprotein E in plasma and the HDL fraction were significantly correlated with the degree of CSO-PVS, indicating that low apolipoprotein E content in HDL may increase the risk of PVS. However, Evans et al. (49) recently found that increased HDL-C levels were associated with a more severe PVS burden in the hippocampus, CSO, and midbrain regions but not in the BG region. These contradictory conclusions may arise from differences in study design, population characteristics, and control of confounding variables. This study introduces the novel inflammatory metabolic indicator UHR for the first time, finding that higher UHR levels were independently associated with an increased risk of moderate-to-severe PVS. This finding supports the complex involvement of metabolic-inflammatory imbalance in PVS. Furthermore, UHR has the potential to serve as a biomarker for assessing the risk of moderate-to-severe PVS, providing substantial support for its diagnosis. Additionally, given the lack of standardized clinical thresholds for UHR in our specific patient population, we objectively and systematically stratified patients used quartile grouping. This approach allows us to avoid the potential pitfalls of arbitrary threshold setting while ensuring an even distribution of participants across UHR groups, which enhances the statistical power of our findings. This is also consistent with previous studies in cardiovascular and cerebrovascular domains, which have effectively utilized quartile or quintile groupings to delineate UHR levels among patient cohorts (21, 32, 50–52).

Currently, UHR has been established as a reliable inflammatory indicator, and its role in metabolic inflammatory diseases has attracted increasing attention. UHR reflects the balance between the antioxidant effect of UA and the anti-inflammatory effect of HDL-C (53). An increase in this ratio indicates elevated oxidative stress and inflammatory response in the body, which aligns closely with the pathological mechanism of PVS, where the imbalance between reactive oxygen species (ROS) generation and the antioxidant defense mechanism can lead to cellular damage and vascular endothelial dysfunction. Although direct evidence of a causal association between UHR and PVS is lacking, UA and HDL-C, as core components of UHR, provide important insights into this potential relationship. UA mitigates the generation of free radicals by scavenging ROS and chelating metal ions, thereby protecting cells from oxidative damage and exerting a neuroprotective effect (54). However, excessive UA accumulation can cause its antioxidant properties to shift into a pro-oxidant effect, thereby contributing to PVS: (1) It promotes inflammation through enhancing ROS generation, activating NLRP3 inflammasome, and stimulating pro-inflammatory signaling (55–57); (2) It triggers the peroxynitrite anion-mediated lipid oxidation while reducing the production and bioavailability of nitric oxide in endothelial cells, exacerbating vascular endothelial dysfunction (58). (3) It increases BBB permeability (59), aggravating the formation of PVS. These mechanisms induce vascular endothelial injury, matrix remodeling, and impaired fluid drainage, which collectively promote the development of PVS (60). Conversely, HDL-C possesses anti-inflammatory and antioxidant properties that can counteract the damaging effects of elevated UA. However, in cases where the UHR is elevated, the protective role of HDL-C may be compromised, particularly in the presence of chronic inflammation often linked with high LDL-C levels (61). This interplay between elevated UA and dysfunctional HDL-C may exacerbate endothelial dysfunction and promote the expansion of PVS. Further investigation into these complex interactions is warranted, as elucidating the underlying biological mechanisms could provide valuable insights into therapeutic strategies aimed at managing PVS and related cerebrovascular conditions.

This study also evaluated the association between UHR and PVS burden across different brain regions. The results revealed a linear relationship between UHR and both moderate-to-severe BG-PVS burden and moderate-to-severe CSO-PVS burden. In the categorical model for UHR, the Q2, Q3, and Q4 groups exhibited an increased risk of moderate-to-severe BG-PVS compared to the Q1 group, whereas only the Q4 group demonstrated an increased risk of moderate-to-severe CSO-PVS. One possible explanation is the anatomical differences in PVS structures across various brain regions. The lenticulostriate and thalamostriate arteries in the BG region are surrounded by a double-layered pia mater, enabling the subpial space to communicate directly with the subarachnoid space. As a result, PVS in the BG region may be more susceptible to systemic metabolic indicators. Conversely, the penetrating superficial and cortical arteries leading to the CSO region are covered by a single layer of pia mater, with the subpial space in this area connecting indirectly to the subarachnoid space. This relatively closed drainage structure may limit the influence of peripheral metabolic factors on CSO-PVS; however, it may also render this region more vulnerable to reduced clearance efficiency. Furthermore, the PVS burden in different regions may involve distinct pathophysiological processes. For example, BG-PVS is associated with hypertensive arterial lesions, while CSO-PVS is more closely linked to CAA (62). Endothelial dysfunction related to hypertension and systemic inflammation disrupts the BBB, leading to a series of reactions, including plasma protein leakage into the PVS, impaired vasodilation, myelin formation and repair disorders, and obstruction of perivascular fluid drainage, which promote PVS formation in the BG region (63). Consistently, numerous studies have found a strong correlation between elevated UHR levels and hypertension (64, 65). It is hypothesized that UHR can exacerbate the oxidative stress cascade, mediating damage to hypertensive cerebral arteries, which results in decreased pulsatility in perforating arteries and diminished clearance efficiency of the glymphatic system. According to the clearance hypothesis, CSO-PVS indicates chronic poor drainage around pial arterial vessels, making individuals more susceptible to amyloid protein clearance defects and the development of CAA (66). Lipid metabolism may also play a role in the occurrence of CAA. The apolipoprotein E ε4 gene is a genetic risk factor for CAA, influencing the conformation and clearance of amyloid-*β* (67). It is posited that increased UHR levels may decrease apolipoprotein-dependent amyloid-β clearance efficiency, exacerbating CSO-PVS.

Furthermore, this study evaluated the association between UHR and the moderate-to-severe PVS burden in different subgroups. The results suggested that the association between UHR and the moderate-to-severe PVS burden remained significant in most subgroups, except for the subgroup with LDL-C < 3.4 mmol/L. Notably, our interaction analysis identified a significant interaction effect between LDL-C levels and UHR, suggesting that the relationship between UHR and PVS burden may vary depending on LDL-C levels. This finding underscores the importance of considering lipid profiles when evaluating the effects of UHR on vascular health. Mechanistically, elevated LDL-C is known to contribute to the development of atherosclerosis, leading to endothelial dysfunction and impaired vascular homeostasis. Such conditions may exacerbate the negative effects of increased UHR on the PVS burden. Specifically, high LDL-C levels can promote oxidative stress and inflammation within the vascular system, which could diminish the protective effects of HDL-C and increase susceptibility to vascular damage. Research suggests that the LDL-C to HDL-C ratio is a critical indicator of cardiovascular health, where a higher ratio correlates with an increased risk of atherosclerotic events and poorer vascular outcomes (68). Additionally, the antioxidant and anti-inflammatory properties of HDL-C can be compromised in the presence of elevated LDL-C levels, leading to impaired endothelial repair mechanisms (61). Therefore, when LDL-C levels are elevated, the protective effects of HDL-C on vascular structures may be diminished, amplifying the detrimental effects associated with high UHR. This dynamic interaction may elucidate the observed association between UHR and PVS burden, emphasizing the need for future studies that explore these interrelationships further to provide enhanced context for lipid management strategies in patients at risk for cerebrovascular diseases.

Previous studies have confirmed that UHR has a certain predictive effect on chronic inflammatory diseases such as coronary artery stenosis, prediabetes, and sacroiliitis (69–71), suggesting that UHR can serve as a convenient, accessible, and non-invasive clinical assessment tool. In our study, the incremental predictive value of UHR for the moderate-to-severe PVS burden was evaluated using the NRI and IDI, which indicated that the predictive performance for the moderate-to-severe PVS/BG-PVS/CSO-PVS was significantly improved after introducing UHR into the basic model. Finally, sensitivity analyses revealed that log2UHR was independently associated with the moderate-to-severe PVS burden. After excluding the patients who had received lipid-lowering drugs, the association between UHR and the moderate-to-severe PVS burden remained significant. This further verified the robustness of the association between UHR and PVS burden.

The clinical utility of UHR in evaluating cerebrovascular disease risk is promising, particularly as a simple and cost-effective biomarker that can supplement existing diagnostic practices. Given the association between UHR and the burden of PVS, incorporating UHR measurements could enhance risk stratification for patients presenting with symptoms indicative of cerebrovascular conditions or those with established risk factors such as hypertension, diabetes, and dyslipidemia. To facilitate its integration into clinical workflows, routine UHR evaluation could be considered as part of the standard metabolic panel during initial patient assessments. Specifically, clinicians could utilize UHR alongside traditional risk factors to evaluate patients’ vascular health more comprehensively. By establishing thresholds for UHR that correlate with increased PVS burden, clinicians can identify individuals who may require more frequent monitoring, referrals for imaging studies, or targeted therapeutic interventions.

The study, however, is not without limitations. Primarily, it focused on assessing the PVS burden in the BG and CSO regions, neglecting less common distribution areas like the cerebellum, corpus callosum, and hippocampus. Future research should comprehensively examine PVS distribution across the entire brain to thoroughly explore the relationship between UHR and the spatial specificity of PVS burden. Secondly, due to the limited sample size, there may be insufficient statistical power, potentially weakening the observed associations. Thirdly, this is a single-center study with all patients recruited from a single hospital in China. The clinical characteristics, ethnic backgrounds, and health system practices of the study population might not be representative of broader populations, thereby affecting the generalizability of our findings. Future research involving multicenter designs and diverse populations is essential to validate these findings and enhance their applicability to various clinical settings. Fourthly, while multiple confounders have been adjusted for in our analyses, it is important to recognize that other unaccounted or unmeasured confounders may still exist. These variables could potentially influence the observed association between UHR and PVS burden, warranting caution in the interpretation of our findings. Lastly, the study is a cross-sectional analysis, limiting our ability to establish a causal relationship between UHR and PVS burden. Additionally, the temporal sequence between UHR levels and PVS burden remains unclear. More extensive longitudinal studies are warranted to better understand how UHR influences the onset and advancement of PVS.

In conclusion, this study provides novel insights into the role of UHR in the burden of PVS in the brain. The findings reveal a positive association between UHR levels and the risk of moderate-to-severe PVS burden. Notably, the association was consistent across different brain regions, including the BG and CSO, and remained robust in subgroup analyses. The inclusion of UHR significantly improved the predictive performance for PVS burden, underscoring its potential as a non-invasive, accessible biomarker for assessing PVS severity. Moreover, the study highlights the complex interplay between metabolic-inflammatory factors and PVS formation, emphasizing the need for further research to elucidate the underlying mechanisms and clinical implications of UHR in PVS-related neurological disorders. Future studies should focus on larger, longitudinal cohorts to validate these findings, establish causality, and explore the therapeutic implications of targeting UHR in the management of PVS-associated conditions.

## Data Availability

The raw data supporting the conclusions of this article will be made available by the authors, without undue reservation.
